# Exposure to attachment narratives dynamically modulates cortical arousal during the resting state in the listener

**DOI:** 10.1002/brb3.1007

**Published:** 2018-06-06

**Authors:** Viola Borchardt, Galina Surova, Johan van der Meer, Michał Bola, Jörg Frommer, Anna Linda Leutritz, Catherine M. Sweeney‐Reed, Anna Buchheim, Bernhard Strauß, Tobias Nolte, Sebastian Olbrich, Martin Walter

**Affiliations:** ^1^ Clinical Affective Neuroimaging Laboratory Magdeburg Germany; ^2^ Department of Behavioral Neurology Leibniz Institute for Neurobiology Magdeburg Germany; ^3^ Clinic for Psychiatry and Psychotherapy University of Leipzig Leipzig Germany; ^4^ Laboratory of Brain Imaging Neurobiology Center Nencki Institute of Experimental Biology of Polish Academy of Sciences Warsaw Poland; ^5^ Clinic for Psychosomatic Medicine and Psychotherapy University Clinic Magdeburg Magdeburg Germany; ^6^ Clinic for Psychiatry and Psychotherapy Otto von Guericke University Magdeburg Magdeburg Germany; ^7^ Neurocybernetics and Rehabilitation Department of Neurology and Stereotactic Neurosurgery Otto von Guericke University Magdeburg Germany; ^8^ Institute of Psychology University of Innsbruck Innsbruck Austria; ^9^ Institute of Psychosocial Medicine and Psychotherapy University Hospital Jena Jena Germany; ^10^ Anna Freud National Centre for Children And Families London UK; ^11^ Wellcome Trust Centre for Neuroimaging University College of London London UK; ^12^ Clinic for Psychiatry, Psychotherapy and Psychosomatic University Clinic Zurich Zurich Switzerland; ^13^ Clinic for Psychiatry and Psychotherapy Eberhard‐Karls University Tuebingen Germany

**Keywords:** affective stimulation, attachment, EEG, human social interactions, resting state, vigilance

## Abstract

**Background:**

Affective stimulation entails changes in brain network patterns at rest, but it is unknown whether exogenous emotional stimulation has a prolonged effect on the temporal dynamics of endogenous cortical arousal. We therefore investigated differences in cortical arousal in the listener following stimulation with different attachment‐related narratives.

**Methods:**

Resting‐state EEG was recorded from sixteen healthy subjects for ten minutes each with eyes closed: first at baseline and then after passively listening to three affective narratives from strangers about their early childhood experiences (prototypical for insecure‐dismissing, insecure‐preoccupied, and secure attachment). Using the VIGALL 2.1 algorithm, low or high vigilance stages in consecutive EEG segments were classified, and their dynamic profile was analyzed. Questionnaires assessed the listeners’ emotional response to the content of the narrative.

**Results:**

As a general effect of preceding affective stimulation, vigilance following the stimulation was significantly elevated compared to baseline rest, and carryover effects in dynamic vigilance profiles were observed. A difference between narrative conditions was revealed for the insecure‐dismissing condition, in which the decrease in duration of high vigilance stages was fastest compared to the other two conditions. The behavioral data supported the observation that especially the insecure narratives induced a tendency in the listener to affectively disengage from the narrative content.

**Discussion:**

This study revealed carryover effects in endogenous cortical arousal evoked by preceding affective stimulation and provides evidence for attachment‐specific dynamic alterations of brain states and individual differences in emotional reactivity.

## 1 INTRODUCTION

To navigate daily life successfully, humans need the capacity to allocate attentional resources in response to environmental cues (Skarratt, Cole, & Kuhn, 2012). The human brain constantly integrates and interprets input from sensory modalities, rapidly reacts to changes in the environment, and adapts its response accordingly. These response patterns usually entail a broad intersubject variability. Furthermore, these patterns are, among other influences, subject to variations in the individual’s mood, motivation, arousal, and fatigue (Bola & Borchardt, 2016). Upon processing of a cognitive or affective stimulus, brain states can be subject to a lasting influence of this stimulus, which induces carryover effects, and thus, the brain’s baseline activity is transiently altered until it is gradually restored over time (Barnes, Bullmore, & Suckling, 2009).

The brain’s dynamic response to the perception of a situation can furthermore be shaped by the individual’s past experiences (Davidson, 1998), especially when processing interpersonal or social information (Buchheim, George, Gündel, & Viviani, 2017). Attachment theory, a developmental psychology framework, suggests that based on experiences in early childhood, such as those involving interactions between a child and his or her primary caregivers, an “internal working model” (IWM) of relational expectations is built. This IWM underpins social–emotional functioning and shapes how the child establishes and maintains social bonds, as well as behavior and regulation of affect in interpersonal relationships later in life (Cassidy, 2008; Nolte, Guiney, Fonagy, Mayes, & Luyten, 2011). In brief, a secure attachment representation is based on an affirmative interaction between child and parents. The caregiver is responsive to the child’s needs, which results in the development of a positive model of self and others, linked with supportiveness and trustworthiness (Vrticka & Vuilleumier, 2012). In contrast, insecurely attached individuals tend to have experienced unresponsive or inconsistently behaving attachment figures and develop attachment anxiety or avoidance (Vrticka & Vuilleumier, 2012). These attachment patterns in children can be discerned with the “Strange Situation Test” (Ainsworth, Blehar, Waters, & Wall, 1979), where the baby is placed into separated and united situations with the mother and strangers. While securely attached infants show a balance between exploration and bonding behavior, insecure‐dismissing babies avoid or ignore the caregiver due to their experiences of past inadequate responses to their needs. They show a hypoactivation of the attachment system. Insecure‐ambivalent/preoccupied individuals, in contrast, show a hyperactivation of the attachment system as a response to unpredictable caregiving, which results in low capabilities of exploration behavior and high distress, when the caregiver disappears. The IWM further influences mental and physical health in later life, and thus, an insecure attachment can be seen as a risk factor for the development of mental disorders (Cassidy, Jones, & Shaver, 2013; Strauss & Brenk‐Franz, 2016). In adults, different attachment representations also are associated with characteristic speech patterns when asked to reflect upon early experiences, and these discourse features are known to influence the counterpart in a social interaction (e.g., Martin, Buchheim, Berger, & Strauss, 2007). An investigation of the influence of attachment representations on subsequent affective processing revealed carryover effects of prototypical narratives characteristic of different attachment representations. These differentially impacted on self‐reported well‐being and the interpersonal disposition of the listener (Kirchmann, Thomas, Brüderle, & Strauß, 2011; Martin et al., 2007).

Affective information processing can, furthermore, be influenced by internally stored representations of stimuli, ideas, or experiences that are called “latent schema.” Schemas are patterns of thought and behavior including internal representations of oneself, others, and the environment. They are activated by certain environmental events and shape how stimuli are perceived in a particular context (Krause et al., 2016; Nolte et al., 2011). Adverse experiences occurring early in life might lead to the development of negative schemas, which can simultaneously contribute to and be exacerbated by depressive symptoms (Disner, Beevers, Haigh, & Beck, 2011). For example, according to the cognitive model of depression (Beck, 1987), activation of negative schemas can be seen as a psychopathologically relevant carryover effect of an environmental trigger and may bias subsequent information processing (Disner et al., 2011). A link between dysphoric mood and dismissing attachment style has been localized in the precuneus and in the supplementary motor area—a brain region that has been implicated in empathy (Fan, Duncan, de Greck, & Northoff, 2011; Yaseen, Zhang, Muran, Winston, & Galynker, 2016). Further evidence for an interaction between insecure attachment and depression severity has been observed in cortico‐striato‐thalamic circuits of affect regulation (Galynker et al., 2012). Previous research based on resting‐state functional magnetic resonance imaging (rs‐fMRI) has suggested that processing of emotional stimuli with attachment‐related content induces carryover effects and alters brain network configurations (Borchardt et al., 2015; Krause et al., 2016).

These carryover effects are likely to be related to markers of cortical arousal. Co‐occurrence of arousal‐related changes in brain states has been shown in simultaneous fMRI and EEG (Chang et al., 2013, 2016; Critchley, 2009; Olbrich et al., 2009; Sadaghiani, Hesselmann, Friston, & Kleinschmidt, 2010; Sadaghiani et al., 2012), which supports a direct investigation of arousal markers in EEG.

As a marker of cortical arousal, different stages of vigilance can be differentiated. Based on EEG acquired during the awake resting state, it is possible to objectively assess and differentiate stages of cortical arousal as well as their trends over time. Stages of high cortical arousal are characterized by tonic alertness, fast responsiveness, and maintained external attention. In contrast, reduced external awareness, drowsiness, and sleepiness describe stages of low cortical arousal (Sander, Hensch, Wittekind, Böttger, & Hegerl, 2015). Individual regulatory processes have been shown to create activity to stabilize or reduce vigilance, dependent on whether the environment is more or less stimulating (Hegerl, Himmerich, Engmann, & Hensch, 2010; Hegerl, Sander, Olbrich, & Schoenknecht, 2009). Here, the term cortical arousal refers to neurophysiological processes and the degree of activation of the central nervous system. For the remainder of this text, we will refer to the term vigilance as tonic neurophysiological cortical arousal during an awake state (Hegerl & Hensch, 2014; Oken, Salinsky, & Elsas, 2006). We will further use the term “stages of vigilance” to refer to distinct global functional brain states (Hegerl, Wilk, Olbrich, Schoenknecht, & Sander, 2012). Previous research in healthy subjects has revealed that over a 30‐min resting state, firstly vigilance shifts from higher to lower stages over time and, secondly, the amount of time spent in lower stages is longer (Olbrich et al., 2009).

Although vigilance trends have shown considerable interindividual differences, it is intra‐individually relatively stable (Van Dongen, Baynard, Maislin, & Dinges, 2004). Trait aspects of cortical arousal, such as the degree of arousal instability and the speed of vigilance decline, were confirmed to be temporally stable in a test–retest comparison (Huang et al., 2015).

Despite little being known about whether or how emotional stimuli influence cortical arousal in the course of affective information processing, the investigation of trends in cortical arousal is crucial for the separation of vigilance‐associated signal changes from those specifically related to affective processing. While it has been shown that affective stimulation entails changes in brain network patterns at rest (Borchardt et al., 2015; Eryilmaz, Van De Ville, Schwartz, & Vuilleumier, 2011; Krause et al., 2016), it has not yet been investigated whether exogenous emotional stimulation has a prolonged effect on the temporal dynamics of endogenous cortical arousal.

### 1.1 Aim

To reveal how emotional reactivity influences transient functional brain states, we investigated differences in cortical arousal induced by listening to narratives that are prototypical of the three main attachment representations at a high temporal resolution using EEG. These specific audio stimuli have been chosen as they represent attachment‐specific socio‐affective cues, which are assumed to evoke different emotional reactions in the narrator’s counterpart. We hypothesized that processing of socio‐affective information contributes to the induction of transient changes in brain states to different degrees dependent on attachment‐specific discourse features. We expected to find a general increase in vigilance during resting states following affective stimulation as an effect of preceding stimulation. Examining the dynamic patterns of cortical arousal after the offset of an affective stimulus may provide information to aid understanding of neuronal processes during emotional reactivity. In an important manner, we sought to investigate attachment‐specific differences in cortical arousal elicited by emotional reactivity of the listener to the discourse features of the narratives.

## 2 METHODS

### 2.1 Subject cohort

Thirty healthy right‐handed, native German speakers were screened for the absence of any psychiatric, neurological, or medical illness and included if the common exclusion criteria for magnetic resonance imaging were not met. The institutional review board of the University of Magdeburg, Germany, approved the study, and all subjects provided written informed consent.

### 2.2 Data acquisition

Simultaneous resting‐state EEG and fMRI data were acquired in a 3T Siemens Verio MRI scanner (Siemens, Erlangen, Germany) with a 12‐channel receiver head coil. The setup of the EEG is equivalent to that described in van der Meer et al. (2016): EEG signals from an MR‐compatible EEG cap (Braincap MR, Brain Products, Gilching, Germany) with 32 channels were recorded according to the 10–20 system. All electrodes were referenced to the FCz position with a ground electrode located at the AFz position. The impedance of all electrodes was maintained below 10 kΩ throughout the recording. Four carbon wire loops (CWLs) were sewn onto the outer surface of the EEG cap at left‐frontal, left‐posterior, right‐frontal, and right‐posterior locations. In addition, two CWLs were attached to the cables connecting the EEG cap to the EEG amplifier (BrainAmpMR Plus). Motion was minimized using soft pads fitted over the ears, and participants were given earplugs to minimize noise.

### 2.3 Experimental design

**Figure 1:**
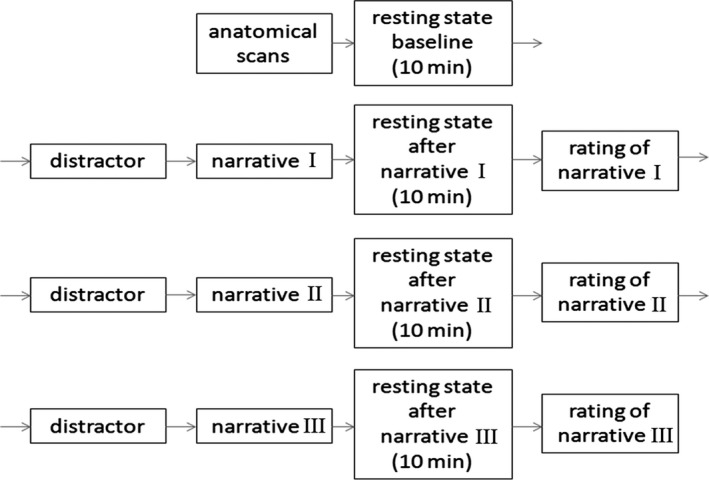
Experimental design of the study

After participants were outfitted with the EEG cap, they performed a rest–task–rest paradigm in the MR scanner (Figure 1).

First, a baseline resting state was measured, during which subjects were instructed to lie still with their eyes closed, to think of nothing in particular, and to stay awake. Each of the subsequent three blocks consisted of four parts. First, the subjects were asked to perform easy calculations for 90 s as a distractor task. The subjects then listened to one of three affective narratives about experiences in the early childhoods of strangers, which was directly followed by a 10‐min post‐task resting‐state MRI scan. During the resting state following exposure to one of the narratives, the subjects continued to lie still with their eyes closed. Subjects were further instructed to think about the content of the narrative. In this way, we aimed to capture the subject’s emotional response in the absence of any particular task to regulate affect. Afterwards, subjects were asked to rate both their own feelings and the narrative with the questionnaire described below. This procedure was iterated, such that each narrative was presented once. To control for effects of presentation order, the sequence of narratives was randomized between subjects.

The narratives were prototypical excerpts of the Adult Attachment Interview (AAI), reliably coded and selected (by AB), in which the interviewees describe their relationship with their father and mother during early childhood with characteristic adjectives and specific examples (Hesse, 2008). Narratives with the AAI categories secure, insecure‐dismissing, and insecure‐preoccupied were selected from a sample of interviews with patients with anxiety disorders. To ensure anonymity and to avoid the impact of different voices, all transcripts were authentically recited while mimicking the original discourse characteristics (by AB). The narratives have been validated in previous studies (Kirchmann et al., 2011; Martin et al., 2007). To control for length of auditory input, the original insecure‐preoccupied and secure narratives were shortened (insecure‐preoccupied: 4:58 min, secure: 4:08 min, insecure‐dismissing: 3:27 min). In doing so, prosody, attachment‐specific speech patterns, and content were retained, because differences in the form of discourse exist and they are independent of the emotional content. For example, the insecure‐dismissing narrative is excessively brief and restricted in coherence and content, whereas the insecure‐preoccupied narrative is incoherent, embellished, excessively long, and includes information that is irrelevant to the discourse task and unfinished sentences. The secure narrative presents a coherent, autonomous discourse including rich language and vivid examples. The narratives differed in the affective valence of the described interpersonal encounters: The secure and insecure narratives fall into the categories of positive and negative valence, respectively.

### 2.4 Questionnaires

After each rest following a narrative, subjects answered two psychological questionnaires about how they related to the speaker in the narrative to assess emotional reactivity and to compare the behavioral responses between narratives.

The first questionnaire assessed the participant’s feelings toward their counterpart (based on Mertens’ theoretical model of unconsciously elicited relational responses) (Mertens, 2005). Sample items are as follows: “Would you like to be friends with this person?” and “Can you imagine how this person is feeling?” The questions assess the context‐specific attitude of the listener to relate to the narrator. Participants rated the sixteen items with “rather yes,” “rather no,” or “neither yes nor no” on a 3‐point Likert scale. The scores range from 0 to 46, where high scores indicate a high tendency for social interaction and positive countertransference, and low scores indicate negative reactions. The questions assess the context‐specific attitude of the listener to relate to the narrator. The internal consistency of this scale was *α* = 0.87 (Martin et al., 2007).

The second questionnaire was the 8‐item subscale “friendly” from the Impact Message Inventory (IMI; Fingerle, 1998), which is related to the interpersonal circumplex model and assesses an individual’s reaction when being confronted with a particular person (“If I were with this person, I would …”). The questionnaire describes interpersonal relationships by assessing the impact of the narrator on the listener. Subjects evaluated their interpersonal reactions to the person listened to on a 4‐point Likert scale (“not at all” to “exactly”). The scores range from 1 to 4, where high scores indicate high estimation of friendliness; that is, the listener feels very friendly toward the narrator. The internal consistency of this scale was *α* = 0.97 (Fingerle, 1998). Data from one subject were excluded, because technical problems arose during recordings in one condition.

### 2.5 Data preprocessing

Data were processed using MATLAB R2012a (http://scicrunch.org/resolver/SCR_001622) and EEGLab v12.0.2.5b (http://scicrunch.org/resolver/SCR_007292) (Delorme & Makeig, 2004). EEG data were corrected for MR gradient artifacts using the Bergen toolbox (Moosmann et al., 2009) which, based on fMRI motion realignment parameters of head movements, identifies artifact volumes with abrupt head movement to reset the MRI gradient artifact template buffer. The data were highpass filtered at 0.5 Hz and lowpass filtered at 125 Hz. To correct for both helium pump and ballisto‐cardiac artifacts, the CWL correction method was used. In short, CWLs act as additional sensors that accurately track movement artifacts related to heartbeat and residual MRI helium pump activity during scanning. These CWL signals can be used to regress these motion artifacts out of the EEG data (van der Meer et al., 2016). To remove additional artifacts, ICA was applied and any component that contained muscle‐, movement‐, sweating‐, or eye‐artifacts, as revealed by visual inspection by an experienced rater (by MB), was excluded from further analysis. The data were then highpass filtered again at 0.5 Hz, lowpass filtered at 70 Hz, down sampled to 250 Hz, and cut to match exactly the duration of the MR acquisition. As part of the VIGALL pipeline, ICA was performed again and another experienced rater (by GS) excluded any remaining artifactual component.

### 2.6 Vigilance estimation

Using the VIGALL 2.1 algorithm, vigilance stages in consecutive EEG segments (each had a duration of 2.4 s) were estimated. The VIGALL classification is based on the distribution of cortical current density activity over four distinct regions of interest (O1, O2, F3, F4). An estimate of the cortical current density is separately computed for the delta/theta and the alpha frequency range using the LORETA method (Pascual‐Marqui, Michel, & Lehmann, 1994). The delta/theta band was set between 3 and 7 Hz. Each subject’s individual alpha frequency was automatically detected, and alpha band was defined as ±2 Hz around the individual alpha frequency. Each segment was classified into one of six different brain states along a wake–sleep continuum (alertness: 0, A1, A2, A3; drowsiness: B1, B2/3; and sleep: C). During relaxed wakefulness, stage A1 is dominated by alpha activity in occipital electrodes O1 + O2, stage A2 is characterized by a shift of alpha activity to central and frontal areas (anteriorization), and stage A3 shows continued frontalization of alpha in F3 and F4 electrodes. Stage B1 is marked by low‐amplitude nonalpha EEG with slow eye movements and stage B2/3 by dominant delta and theta power. Due to the segment length (>1 s), the algorithm could not distinguish between stages B2/3 and C (characterized by sleep spindles and/or K‐complexes), and each segment in question was assigned to the B2/3 stage. Because electrooculography was not available, stages B1 and 0 (reflecting an alert and mentally active state) could not be distinguished, and each segment in question was assigned to the B1 stage. As data were acquired during the resting state, it is unlikely that subjects were in the highest vigilance stage. After a quality check of the fMRI (good MR quality, no incidental findings, head movement <3 mm and 3°), EEG data (impedances <10 kΩ for all channels and absence of artifacts), and vigilance estimation (successful estimation in at least 95% of the segments, absence of only one constant vigilance stage), datasets of 16 subjects (eight males, mean age 27.3 ± 5.9 years) were of sufficient quality and were included in the analysis. If a dataset from one of the four experimental conditions did not meet the quality standards, the respective subject was excluded.

### 2.7 Statistical analysis

EEG time segments were binarized into high (A1, A2, A3) versus low (B1, B2/3) vigilance stages. For each subject, the numbers of segments classified as representing high vigilance stage were summed per condition, and paired t‐tests were calculated to assess differences between conditions. Because of these repeated tests, a Bonferroni correction was applied to the significance level, resulting in a critical level of a = 0.0125 (criterion: *p *< 0.05).

An ANOVA was used to model vigilance trends as a function of time and to test for interactions between condition and time. Ratios between high to low dynamic vigilance stages and their regressions were visualized for a comparison between all conditions. These ratios provide an intuitive visualization of the decay pattern by combining information from both high and low vigilance stages. Ratios were constructed to protect values against influence of artifacts during vigilance estimation. Subsequent statistics were calculated using only the high vigilance stages, because high and low vigilance stages are reciprocal and complement each other.

To allow visualization of the dynamic evolution of vigilance, for each EEG segment, the percentages of subjects in a low or high vigilance stage were plotted. Linear regressions for high vigilance stages were added to compare the slope and intercept between narratives in an ANCOVA (aoctool in MATLAB) using a Bonferroni adjustment to correct for multiple comparisons (multcompare in MATLAB). Differences in behavioral responses to two questionnaires were compared between narratives using Wilcoxon signed‐rank tests.

To test whether narrative ratings (countertransference and IMI, mediators) explain the relationship between listening to narratives (independent variables) and arousal intercept and slope of postnarrative rests (dependent variables), we calculated four mediation models using the PROCESS macro for SPSS (Hayes, 2013).

## 3 RESULTS

### 3.1 Behavioral effects of prototypical attachment narratives

Responses (average, standard deviation, range in [minimum–maximum]) regarding social interaction with the narrator were 10.7 ± 6.9, [3–22] for insecure‐dismissing, 15.6 ± 5.8, [5–22] for the insecure‐preoccupied, and 17.9 ± 6.1, [7–26] for the secure narrative. Responses (average, standard deviation, range in [minimum–maximum]) regarding how friendly the listener feels toward the narrator were 2.5 ± 0.7, [1.75–3.9] for insecure‐dismissing, 2.7 ± 0.6, [1.25–3.6] for the insecure‐preoccupied, and 3 ± 0.4, [2.1–3.7] for the secure narrative. Correlation coefficients between IMI and the countertransference questionnaire were 0.49, 0.29, and 0.39 for the insecure‐dismissing, insecure‐preoccupied, and secure narrative, respectively.

**Figure 2:**
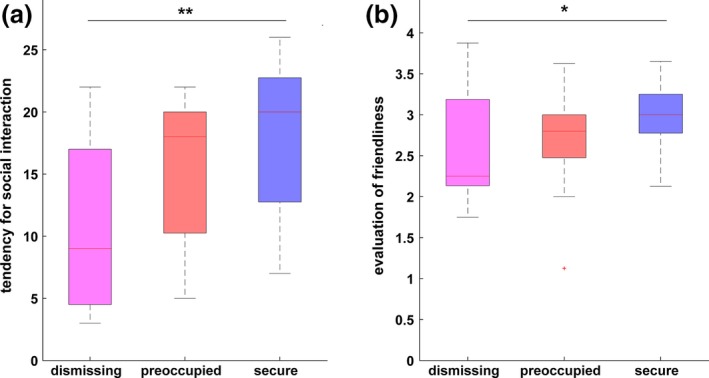
Behavioral effects of narratives. (a) The participants showed the lowest tendency toward potential social interaction following the dismissing narrative. (b) The dismissing narrative was rated as the least friendly. On each box, the central red mark is the median, the edges of the box are the 25th and 75th percentiles, the whiskers extend to the most extreme data points not considered outliers, and outliers are plotted as red crosses. ** indicates *p *< 0.01, * indicates *p *< 0.05

A significant difference in evaluation of friendliness was observed between the reactions to the insecure‐dismissing narrative and the secure narrative (insecure‐dismissing versus secure: *p *= 0.047; Figure 2b), but not between insecure‐preoccupied versus secure (*p *= 0.13) or insecure‐dismissing versus insecure‐preoccupied (*p *= 0.45). This finding was paralleled by the lowest inclination to engage in potential social interaction with the insecure‐dismissing narrator when compared to the secure narrator (*p *= 0.005; Figure 2a). The inclination toward social interaction did not significantly differ between insecure‐preoccupied and secure (*p *= 0.3) and reached trend significance for insecure‐dismissing versus insecure‐preoccupied (*p *= 0.06).

### 3.2 Vigilance trends at baseline

**Figure 3:**
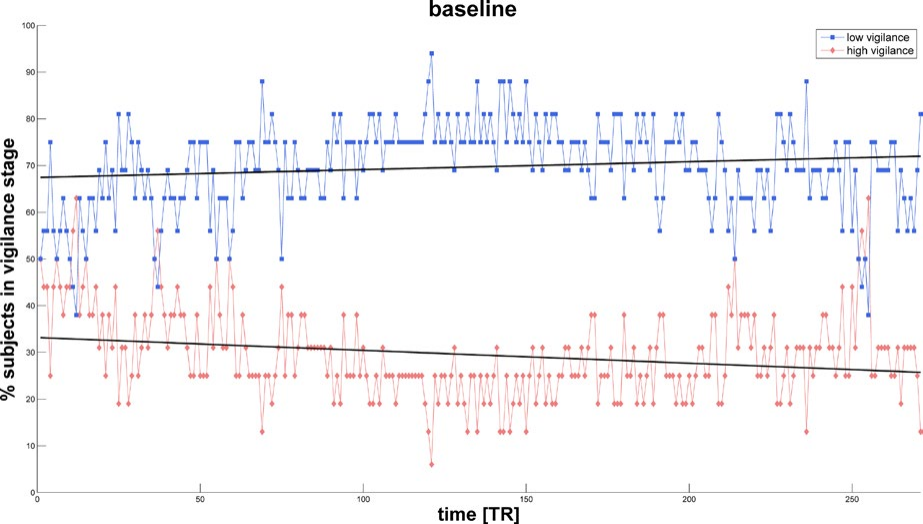
Temporal evolution of the percentage of subjects in high (red) versus low (blue) vigilance stages per TR at baseline. Linear regressions for both high and low vigilance stages are plotted as black lines

At baseline, the percentage of subjects in low vigilance stages was higher than the percentage of subjects in high vigilance stages throughout the recording. The fluctuations in arousal showed no linear trend, although low (high) vigilance followed a mild (inverted) U‐curve, where the midpoint was a maximum (minimum) (Figure 3).

### 3.3 Differences in vigilance level between baseline and attachment narratives

**Figure 4:**
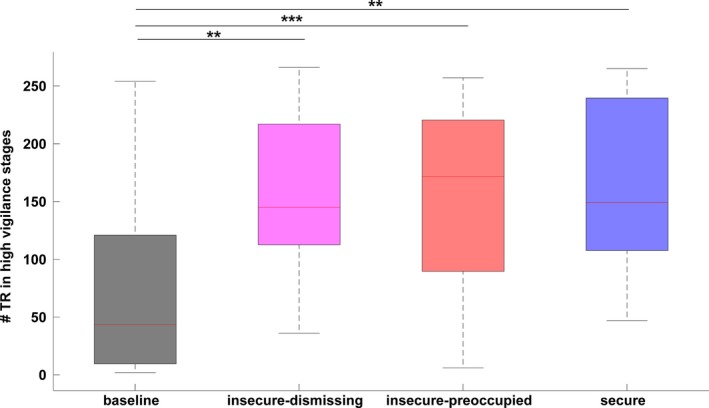
Differences in number of TR time lengths spent in high vigilance stages during the whole EEG timecourse. On each box, the central red mark is the median, the edges of the box are the 25th and 75th percentiles, and the whiskers extend to the most extreme data points not considered outliers. *** indicates *p *< 0.001, ** indicates *p *< 0.01

Following each narrative condition, the time spent in high vigilance stages (quantified as the number of time windows of length TR) increased in comparison with baseline (*a* = 0.0125, *df* = 15). Paired t‐tests (Bonferroni‐corrected for four comparisons, with criterion *p *< 0.05) were applied: baseline versus insecure‐dismissing: *t*(15)  = 3.17, *p *= 0.0063; baseline versus insecure‐preoccupied: *t*(15)  = 4.33, *p *= 0.0006; baseline versus secure: *t*(15)  = 3.98, *p *= 0.0012 (Figure 4).

### 3.4 Dynamic vigilance evolution following exposure to attachment narratives

**Figure 5:**
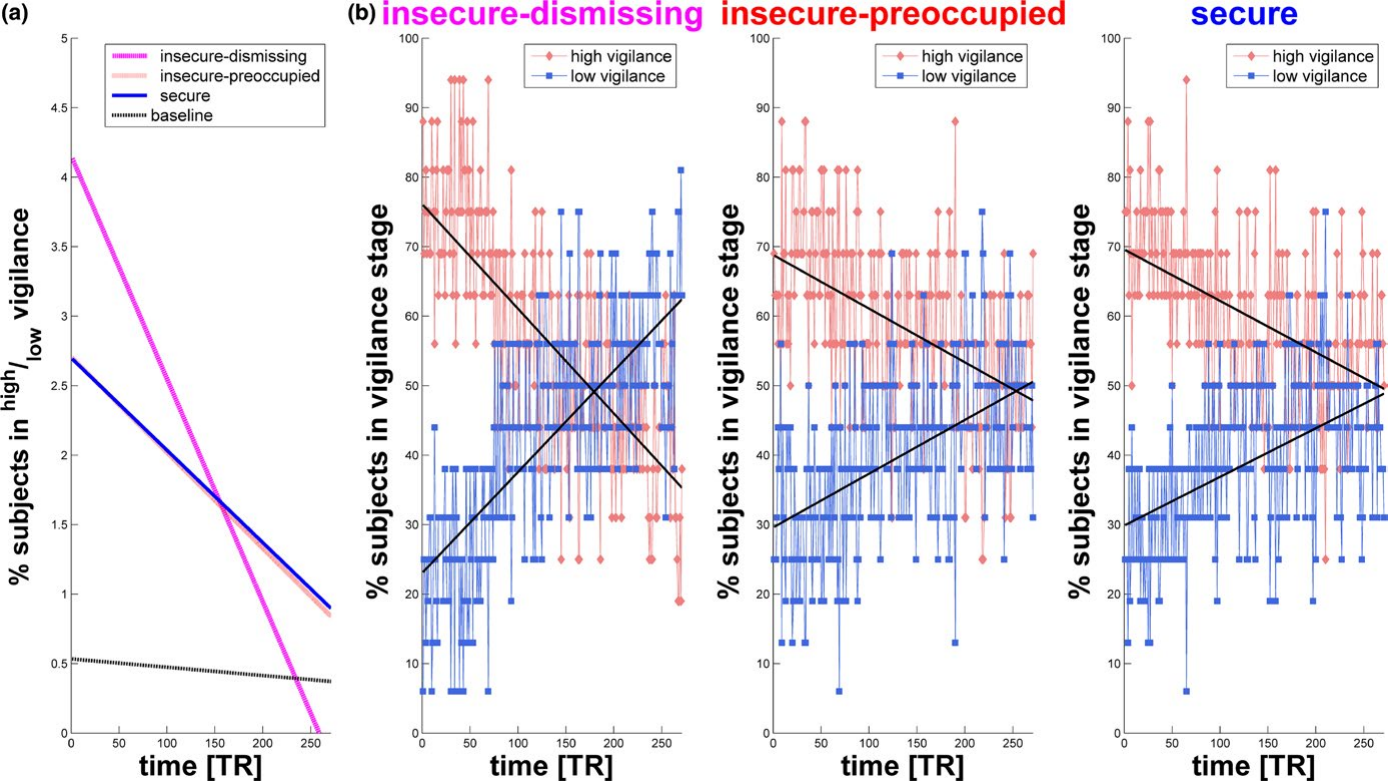
(a) Linear regressions for the ratio of percentage of subjects in high to low vigilance stages between all resting states. (b) Temporal evolution of the percentage of subjects in high (red) versus low (blue) vigilance stages for each TR following the three narratives: insecure‐dismissing (left), insecure‐preoccupied (middle), and secure (right). Linear regressions for both high and low vigilance stages are plotted as black lines. The coefficients for the intercept and slope of the ANCOVA model equations differed between narrative conditions: insecure‐dismissing: y = 76.1−0.151x+ε; insecure‐preoccupied: y = 68.8−0.077x+ε; secure: y = 69.6−0.074x+ε and revealed significant differences between the slopes of the regression in insecure‐dismissing versus insecure‐preoccupied as well as insecure‐dismissing versus secure

An ANOVA revealed a significant interaction between narratives and time (*F*(1,2)  = 29.42, *p *< 0.001). Following the narratives, the percentage of subjects in high vigilance stages was higher than the percentage of subjects in low vigilance stages throughout the recordings. Trends in high and low vigilance stages showed rapid decreases and increases over time, respectively (Figure 5).

Using an ANCOVA, the percentage of subjects in high vigilance stages was modeled as a linear function of time for the three narrative conditions as separate lines.

In the insecure‐dismissing condition, the slope was most negative (while the intercept was higher (n.s.) than in the other conditions), indicating the fastest decrease in high vigilance (Table 1, Figure 5). Post hoc tests following ANCOVA revealed significant differences between the slopes of the regression in insecure‐dismissing versus insecure‐preoccupied as well as insecure‐dismissing versus secure. There was no significant difference between insecure‐preoccupied and secure (Table 2).

**Table 1:** ANCOVA model coefficient estimates for all narrative conditions

Term	Estimate	SE	*T*	*p*
Intercept	71.515	0.724	98.8	<0.001
Dismissing	4.619	1.024	4.51	<0.001
Preoccupied	−2.705	1.024	−2.64	0.0084
Secure	−1.914	1.024	−1.87	0.0619
Slope	−0.101	0.005	−21.82	<0.001
Dismissing	−0.050	0.007	−7.67	<0.001
Preoccupied	0.023	0.007	3.59	0.0004
Secure	0.027	0.007	4.08	<0.001

**Table 2:** Results of the post hoc tests on ANCOVA results

Comparison between two conditions		Estimated difference between slopes	95% confidence interval	
			Lower	Upper
Dismissing	Preoccupied	−0.0734^a^	−0.0999	−0.0469
Dismissing	Secure	−0.0766^a^	−0.1031	−0.0501
Preoccupied	Secure	−0.0032 (n.s.)	−0.0297	0.0233

a *p *< 0.05, and n.s. indicates that only the comparison preoccupied versus secure was not significant at the 0.05 level, because the confidence interval contains 0.0.

None of the four models gave evidence for a significant mediation of the questionnaire scores on the arousal dynamics of the postnarrative rests (Data S1).

## 4 DISCUSSION

This study investigated effects of affective stimulation via prototypical attachment narratives on the listener’s cortical arousal. Following listening to the three different narratives, vigilance increases persisted during subsequent resting periods, while temporal trends in vigilance exhibited different patterns between the narratives. In a specific manner, in comparison with insecure‐preoccupied and secure narratives, listening to the insecure‐dismissing narrative resulted in a reduced inclination to interact with the narrator and led to higher vigilance at the beginning of the subsequent resting state and, at the same time, faster decrease in vigilance.

### 4.1 Vigilance trends at baseline

During the baseline resting recordings, average drowsiness increased while alertness decreased over time (Figure 3), which is an expected physiological effect consistent with previous findings (Olbrich et al., 2009). Furthermore, the pattern of cortical arousal was stable and linear, which is in line with the absence of experimental activation directly before the baseline (Huang et al., 2015; Olbrich et al., 2009; Van Dongen et al., 2004). Indeed, prior to the baseline measurements, subjects had already rested for approximately 15 min.

### 4.2 Dynamic vigilance evolution following affective stimulation

Following affective stimulation, vigilance trends deviated from the stable linear pattern observed at baseline. During the entire resting‐state period following affective stimulation, vigilance was elevated compared to the baseline, indicating a task‐general (i.e., narrative‐type independent) effect (Figure 4). It is conceivable that, in comparison with endogenous mind‐wandering, listening to the narratives is a stimulating event that induces arousal and results in an increase in overall vigilance.

Directly after affective stimulation, the number of subjects in high vigilance stages increased and exceeded that in low vigilance stages. During the course of the resting period, the number of subjects in high vigilance stages decreased and approached the baseline level by the end (Figure 5b). A return to the baseline vigilance level was to be expected given that postnarrative monitoring also took place during a state of rest.

The initial persistence of high vigilance following the narratives points to the existence of carryover effects, which arise when an early experimental condition influences a subsequent one. Here, the prior affective stimulation probably lingers in the subject’s mind. Its aftermath can be seen in the subsequent decrease in high vigilance stages, as vigilance is gradually restored over time to the baseline level and even continues to decrease.

Following the dismissing narrative, the decrease in high vigilance stages over time was fastest (Figure 5), indicating an attachment‐specific effect of preceding exposure to the narrative on subsequent cortical arousal patterns.

As there was no evidence for an increase in vigilance toward the end of the resting‐state periods, we exclude a potential influence of expectation of the subsequent rating on vigilance trends.

### 4.3 Behavioral effects of attachment narratives

The behavioral data support the hypothesis that the three narratives induced different emotional reactions in the listener and that the insecure narratives had a stronger impact than the secure one (Figure 2). The behavioral “countertransference” reactions of listeners toward the insecure‐dismissing narrative were characterized both by the lowest inclination toward social interaction and the lowest rating of friendliness. This, firstly, supports the interpretation that a faster termination of the engagement between the listener and the interviewee occurs following exposure to the insecure‐dismissing narrative and, secondly, is consistent with observations in an earlier study (Krause et al., 2016).

### 4.4 The role of the attachment narratives

Differences in dynamic vigilance trends following the three narratives led to the interpretation that the attachment‐related content influenced listeners differently and triggered different cortical arousal regulation patterns.

Comparing change patterns and behavioral data between narrative conditions showed that the insecure‐preoccupied and secure conditions resemble each other, which may suggest that the content of these two narratives evokes both comparable emotional reactions and carryover effects, namely an increased propensity to interact with the content presented in the narrative.

In contrast, the strongest attachment‐specific effects on trends in cortical arousal were observed in the insecure‐dismissing condition.

This finding is in line with previous research showing that after listening to the insecure‐dismissing attachment narrative, a network related to social aversion was activated and showed increased intranetwork functional connectivity (FC). Moreover, neuronal responses were predicted by the listener’s attachment representation and childhood trauma characteristics, supporting a causal interrelation of brain state changes and subsequent changes in emotional reactivity. Interactions of nodes within the social aversion network with both the dorsolateral prefrontal cortex and parietal cortex were mediated by the individual’s attachment anxiety and evaluation of friendliness (Krause et al., 2016).

Previously, we applied graph theory to fMRI data and identified a brain network including the supplementary motor area (SMA) that decreased its FC specifically following listening to the insecure‐dismissing narrative. This finding indicated differences in brain state changes according to emotional reactivity between conditions (Borchardt et al., 2015).

In an interesting manner, in the insecure‐dismissing condition, the degree of FC of the SMA approached baseline values fastest compared to the other conditions. Furthermore, a similar characteristic temporal profile was observed following listening to the insecure‐dismissing narrative in the present study, in that initial hyperarousal decreased faster below baseline level.

Taken together, the results of the current study investigating dynamic vigilance trends match those observed in our previous study investigating dynamic brain network connectivity patterns and both consistently showed an attachment‐dependent effect of the preceding affective stimulation with an insecure‐dismissing narrative on temporal profiles of brain states. Thus, vigilance state changes seem to converge with previously observed alterations in fMRI brain state dynamics.

Given that negative stimuli impact upon subsequent processing for a longer duration than positive stimuli do (Morriss, Taylor, Roesch, & van Reekum, 2013)*,* one may hypothesize that due to the processing of the discourse characteristics, the insecure‐dismissing narrative causes early and intensive ruminative thoughts in the listener, which would produce immediate alterations in the brain state patterns typically observed in the resting state. However, if this were the case, continuously elevated vigilance stages would be to be expected. The evident vigilance decay is in conflict with that hypothesis.

The specific discourse characteristics of attachment representations are known to influence the counterpart. Social interaction with an individual with an insecure‐dismissing attachment representation has been shown to induce feelings of distance in the counterpart, that is, listeners’ evaluations of friendliness and their readiness for social interaction were lowest (Kirchmann et al., 2011; Krause et al., 2016; Martin et al., 2007). The fast vigilance decay effect combined with the observation of earlier faster network reconfiguration back to a baseline level may be thus interpreted as a freeing from the stimulus content. One may speculate that the dismissing narrative prompted the listener to rapidly disengage from the stimulus content; hence, the vigilance‐enhancing effect of the narrative vanished and led to a fast decline of vigilance. The carryover effects induced by narratives characteristic of an insecure‐dismissing attachment representation exhibited a specific reactivity pattern, which seems to be indicative of a “functional disengagement” from someone else’s unpicturesque biographical experiences presented with the discourse characteristics that accompany this type of attachment narrative. Such disengagement may be seen as a dispirited process with the attempt to free one’s own feelings and to shift attention away from attachment events that are still lingering in one’s mind. The fast disappearance of the carryover effect in the dismissing condition may be interpreted as a blunted emotional reaction in the listener, with a consequently faster termination of the entanglement between the listener and the interviewee.

### 4.5 Importance for some aspects of interpersonal phenomena in psychopathology

Investigating how individual responses of healthy humans result in shifts in cortical arousal levels could serve as a useful comparison to compromised emotional recovery that exists in clinical populations. Compared to a healthy population, patients with conditions or pathologies associated with disturbances in social–emotional functioning display an over‐representation of insecure attachment, and they often show pronounced attachment anxiety or avoidance (Dozier, Stovall‐McClough, & Albus, 2008; Juen, Arnold, Meissner, Nolte, & Buchheim, 2013).

Patients suffering from clinical syndromes such as depression, attention deficit hyperactivity disorder, bipolar disorder, and mania have been shown to exhibit distinct patterns of brain arousal regulation (Hegerl et al., 2012).

Therefore, it is likely that a patient’s emotional reactivity might be characterized by altered functional brain network patterns and cortical arousal. For example, depressed patients typically show a hyperstable regulation of vigilance with tonic hyperarousal (Hegerl et al., 2012; Olbrich et al., 2012). Hyperstable vigilance regulation is characterized by more time spent in high vigilance stages, associated with reduced and delayed declines to lower vigilance stages, and fewer switches between vigilance stages during rest (Hegerl & Hensch, 2014). According to the vigilance model of affective disorders, hyperstable vigilance regulation is in line with prolonged sleep onset latency, inner restlessness, and tension. Thus, hyperstable vigilance regulation may be due to an increased tendency to ruminate in depressive patients.

Future studies could investigate whether alterations in vigilance are related to emotional reactivity in patients suffering from conditions known to affect empathy, such as affective disorders and schizophrenia (Besharat & Khajavi, 2013; Kirchmann et al., 2012) and thereby offer potential diagnostic insights leading to improved treatment approaches.

Internal schemata influence an individual’s mode of interaction in everyday relationships, but they are of special interest in therapeutic situations (Harris, 2004). Psychotherapists need to be aware that attachment may influence the therapeutic relationship by shaping transference and countertransference reactions between the patient and the therapist. In particular, encounters with patients having insecure attachment representations may elicit feelings of anger, frustration, or confusion in the therapist (Slade, 1999). With regard to insecure‐preoccupied patients, therapists might feel overwhelmed, because these patients express their needs in a way that challenges and involve the therapists without being able to develop a stable relationship. (Rubino, Barker, Roth, & Fearon, 2000) revealed that insecurely attached therapists showed diminished empathy, particularly with fearful and secure patients. As a whole, responses to insecure‐preoccupied patients tended to be deeper and more empathic than those to the insecure‐dismissing and secure patients. Attachment security has been reported to be a predictor of treatment outcome (Mosheim et al., 2000; Strauss, Lobo‐Drost, & Pilkonis, 1999). Galynker et al. (2012) speculated that depression and attachment insecurity may be subserved by similar affect‐regulating circuits in the brain, which may explain the greater difficulty of treating depression in insecurely attached patients and suggest a contributing role for insecure attachment in depression. If a therapist brings to his mind that countertransference reactions might, for example, lead to disengagement from the patient, success of therapy may be improved. Martin et al. (2007) assessed the influence of patient’s attachment representations on countertransference by playing narratives of patients with different attachment representations to both therapists and medical students. In an interesting manner, the responses from medical students did not differ largely from the therapists, which indicates that the content of the narratives was equally perceived by laypersons and therapists.

### 4.6 Limitations

The small sample size warrants caution, and gender differences could not be analyzed due to even smaller subgroups.

On the behavioral level, attention coincides with high vigilance, because an attentive state of mind requires being in a high vigilance stage. The observed decrease in vigilance probably co‐occurs with deactivation of a subset of brain regions called the frontal–parietal–attention network, whose FC patterns covary with an individual’s state of alertness with the purpose to enable a timely response to stimuli in the environment (Markett et al., 2014; Sadaghiani, Lehongre, Morillon, & Giraud, 2010).

Although we measured EEG and fMRI simultaneously, only the EEG data are presented here, because it appears to be crucial for separating vigilance‐associated brain state changes from those specifically related to cognition. These observations will be included in the interpretation of future BOLD‐based analyses to increase our understanding of the influences of vigilance and the true effects of listening to the emotional narratives.

An advantage of the analysis of EEG data is the low recording cost and the ready availability of EEG equipment in clinical settings.

As we used the CWL correction method to regress motion artifacts related to heartbeat and residual MRI helium pump activity, the EEG data quality is comparable to datasets acquired outside the scanner (van der Meer, 2014).

For future rest–task–rest studies with a similar setup, it would be advisable to shift the anatomical MR scan toward the end of the session to capture the initial arousal regulation patterns on entering the scanner.

## 5 CONCLUSION

Prolonged changes in endogenous cortical arousal following affective stimulation were revealed and provided evidence for narrative‐specific dynamic influences on brain states, which supports the idea of dynamic carryover effects following exogenous affective stimulation. The existence both of characteristic, narrative‐dependent vigilance regulation patterns and corresponding behavioral ratings following the narratives hint at differential influences of the three conditions on emotional reactivity.

Emotional reactivity following listening to the insecure‐dismissing narrative was behaviorally characterized by both the lowest inclination toward social interaction and the lowest rating of friendliness toward the narrator and entailed initial hypervigilance followed by rapid adaptation to the baseline arousal level.

These deviations in emotional reactivity toward counterparts with an insecure‐dismissing attachment representation most likely manifest in an altered social intercourse. Understanding these phenomena is not only relevant to clinical populations with impaired reactions to social stimuli, but could also provide clinicians and psychotherapists with crucial insights into the responses their patients may induce in them and other counterparts.

## CONFLICT OF INTEREST

The authors declare no conflict of interest.
