# Supplementary material for: Exposure to attachment narratives dynamically modulates cortical arousal during the resting state in the listener
Source: Brain Behav. 2018 Jun 6;8(7):e01007. doi: 10.1002/brb3.1007 (PMC6043700; doi:10.1002/brb3.1007)
Supplement: Supplementary file 1 [file BRB3-8-e01007-s001.docx]

**Supplementary material**

**Results of Mediation Analysis**

To test whether narrative ratings (countertransference and IMI, mediators M) explain the relationship between listening to narratives (independent variables X) and arousal intercept and slope of post-narrative rests (dependent variables Y), four mediation models were calculated using the PROCESS macro for SPSS (Hayes, 2013)).

Model 1: X= narrative, Y=slope, M=countertransference.

The direct effect of X on Y was p=0.09.

The total effect of X on Y was p=0.13.

The indirect effect had the confidence intervals [-0.0003, 0.0001] and the Normal theory (Sobel) test was not significant (p=0.62).

Model 2: X= narrative, Y=slope, M=perceived friendliness.

The direct effect of X on Y was p=0.17.

The total effect of X on Y was p=0.13.

The indirect effect had the confidence intervals [-0.0003, 0.0002] and the Normal theory (Sobel) test was not significant (p=0.79).

Model 3: X= narrative, Y=intercept, M=countertransference.

The direct effect of X on Y was p=0.15.

The total effect of X on Y was p=0.27.

The indirect effect had the confidence intervals [-0.0104, 0.1072] and the Normal theory (Sobel) test was not significant (p=0.27).

Model 4: X= narrative, Y=intercept, M=perceived friendliness.

The direct effect of X on Y was p=0.42.

The total effect of X on Y was p=0.27.

The indirect effect had the confidence intervals [-0.0499, 0.0199] and the Normal theory (Sobel) test was not significant (p=0.71).

Using GPower 3.1.9.2 software, the effect size index f^2^ of a linear multiple regression (Fixed model, single regression coefficient, two-tailed, a priori, 1 predictor) of our sample (N=15) was calculated. It is a low effect size of 0.15 with a power of 0.28. There were no significant indirect effects that support a mediating influence of the questionnaire scores on the arousal dynamics of the post-narrative rests.
